# Advances in Geriatric Oncology: Exploring Practical Ways to Optimize Treatment in Older Patients with Cancer

**DOI:** 10.3390/cancers14174129

**Published:** 2022-08-26

**Authors:** Elena Paillaud, Marije E. Hamaker, Pierre Soubeyran

**Affiliations:** 1 Department of Geriatrics, European Georges Pompidou Hospital, Paris Cancer Institute CARPEM, Assistance-Publique Hôpitaux de Paris, F-75016 Paris, France; 2 Clinical, Epidemiology and Ageing, IMRB, INSERM, Université Paris-Est Creteil, F-94010 Creteil, France; 3 Department of Geriatric Medicine, Diakonessenhuis, 3582 KE Utrecht, The Netherlands; 4 Department of Medical Oncology, Institut Bergonié, Inserm U1312, SIRIC BRIO, Université de Bordeaux, F-33076 Bordeaux, France

Cancer is a disease associated with aging, with patients over 70 accounting for 50% of newly diagnosed malignancies and 70% of all cancer deaths. Despite this epidemiologic context, older patients with cancer continue to be underrepresented in clinical trials which are necessary to establish new standards of cancer care. As a result, robust data on the benefit/risk balance for many treatment strategies in these older patients are lacking, putting them at increased risk for treatment toxicity. It is against this background that in this Special Issue and together with CANCERS, we published a series of 25 papers (19 original articles and 6 reviews) looking specifically at exploring practical ways to optimize treatment in older patients with cancer.

The aging of population worldwide, the increasing number of older patients with cancer, and the low inclusion rates of older patients in clinical trials make oncological practice challenging every day. The most common reason for the lack of recruitment of older patients with cancer is selective eligibility criteria, excluding older patients with functional decline, comorbid conditions and/or prior malignancy [1]. While specific guidelines on treating cancer in older patients from scientific societies and systematic review of the literature can help physician in daily practice [2,3,4], these guidelines are not always followed [4]. 

To evaluate vulnerabilities that are not routinely captured in oncology assessments and help physicians select the best cancer treatment, geriatric assessment (GA) prior to initiation of oncologic treatment is recommended. GA is a validated multidimensional tool to assess the overall health status and includes the assessment of domains affecting this population, such as functional status and mobility, psychological health, polypharmacy, comorbidity, nutrition, social support, and cognition. 

Since GA can be time-consuming and requires specific expertise, screening tools such as G8 and modified-G8 have been developed to identify older patients requiring a full GA and multidisciplinary approach. These tools demonstrated high predictive value and performance robustness to detect various definitions of frailty [5]. Moreover, GA can predict cancer treatment toxicity, treatment completion, and survival. The prognostic impact of each GA-domain and their evolution during cancer remain to be clarified and give rise to numerous research works [6,7,8,9]. 

The implementation of GA, currently, is not widespread. The three major impediments to its dissemination are the lack of interdisciplinary collaboration, human resources, and cost. To improve our practice, it is essential to understand the determinants of the collaboration between oncologists and geriatricians [10] and carry out economic evaluations of GA [11]. 

Recent studies have shown that GA-driven interventions can reduced rates of chemotherapy-related toxic effects and treatment modification while increasing treatment completion. However, data regarding the effects of GA-driven interventions on clinical outcomes such as survival rate or quality of life among older patients with cancer are very sparse [12]. A recent randomized study assessed the efficacy of GA-driven interventions and follow-up on six-month mortality, functional, and nutritional status in older patients with head and neck cancer (HNC) and failed to improve these outcomes [13].

A major concern is patient heterogeneity in terms of comorbidities, dependency, or malnutrition (each of these factors being liable to be associated with poor outcomes in the course of cancer treatment). The identification of prognostic factors, including geriatric and oncologic parameters, could help select older patients most likely to benefit from standard treatment. In this Special Issue, several studies identify risk factors for toxicity and/or overall survival in older patients with colorectal carcinoma [14], metastatic pancreatic cancer initiating chemotherapy [15], postoperative endometrial cancer initiating radiotherapy [16], and head and neck cancer undergoing chemoradiation [17]. Other series have shown that routine inflammatory biomarkers such as those incorporated in the Glasgow Prognostic Score (GPS) and the modified Glasgow Prognostic Score (mGPS) [18], the CRP/albumin ratio (CAR) [19], and the B12/CRP index (BCI) [20], may add to the clinical factors required for prognosis evaluation.

The benefit of major cancer surgery among older patients may be limited, and it remains unclear how to optimally select suitable patients. One study showed a high 30-day complication rate and a longer stay on rehabilitation unit after radical cystectomy for selected older patients with muscle-invasive bladder cancer [21]. Based on these risk factors, predictive scores for outcomes have been built such as the pre-operative GRADE or CARG tool, which were evaluated in various studies [22,23].

However, treatment choice also depends on patient’s preference for outcomes, which needs to be assessed explicitly, especially in older patients. In a systematic review, oncologic patients most frequently gave high priority to overall quality of life, followed by overall survival, progression- and disease-free survival, and severe/persistent side effects [24]. The meaning of quality of life varies between persons. A single-center, qualitative interview study assessed how older patients with cancer define quality of life and the components that are most significant to them. Maintaining cognition and independence, staying in one’s own home, and maintaining contact with family and community appear to be the most important aspects of quality of life for older patients with cancer [25].

We hope that the series will prove to be a useful resource for oncologists and geriatricians alike in managing care for their older patients with cancer.
